# Autoimmune Hepatitis Associated With Cryoglobulinemic Vasculitis

**DOI:** 10.14309/crj.0000000000000804

**Published:** 2022-07-05

**Authors:** Pedro Alves da Cruz Gouveia, Maria Teresa de Souza Portela Leal, Sylene Rampche

**Affiliations:** 1 Department of Internal Medicine, Oswaldo Cruz University Hospital, Recife, Brazil; 2 Department of Gastroenterology and Hepatology, Oswaldo Cruz University Hospital, Recife, Brazil

## Abstract

Cryoglobulinemic vasculitis presents liver abnormalities usually associated with hepatitis C. We report a case of a 27-year-old woman with cryoglobulinemia and liver dysfunction secondary to autoimmune hepatitis. The patient developed purpura on the lower extremities and elevated aminotransferases. The investigation of hepatitis C was negative. Autoimmune hepatitis was confirmed by positive ANA, hypergammaglobulinemia, and compatible histological changes. Treatment with prednisone and azathioprine regressed cutaneous vasculitis and decreased aminotransferases. This case describes a rare association of cryoglobulinemic vasculitis and autoimmune hepatitis.

## INTRODUCTION

Cryoglobulins are immunoglobulins that, on exposure to low temperatures, are deposited in small and medium vessels and result in vasculitis. Cryoglobulinemic vasculitis (CV) involves the peripheral nervous system, skin, joints, and kidneys.^1^ Hepatitis C virus (HCV) represents the etiology of CV in approximately 80% of cases. B-cell lymphoproliferative disorders, autoimmune diseases, and other infections are also associated with cryoglobulinemia.^2^

Autoimmune hepatitis (AIH) is an inflammatory process that occurs in the liver mediated by autoantibodies.^3^ The pathogenesis of AIH results from a complex interaction between genetic predisposition, triggering factors, and autoantigens, which cause hepatic infiltration of lymphocytes and plasma cells.^4^ Liver damage in AIH can result in acute liver failure, acute hepatitis, chronic hepatitis, cirrhosis, or hepatocellular carcinoma.^5^

The association of AIH and cryoglobulinemia is rarely described in the literature. We report a patient with CV who presented with liver abnormalities secondary to AIH. This is a challenging diagnosis in patients with CV because the liver damage is closely related to the HCV.

## CASE REPORT

**Figure 1.**
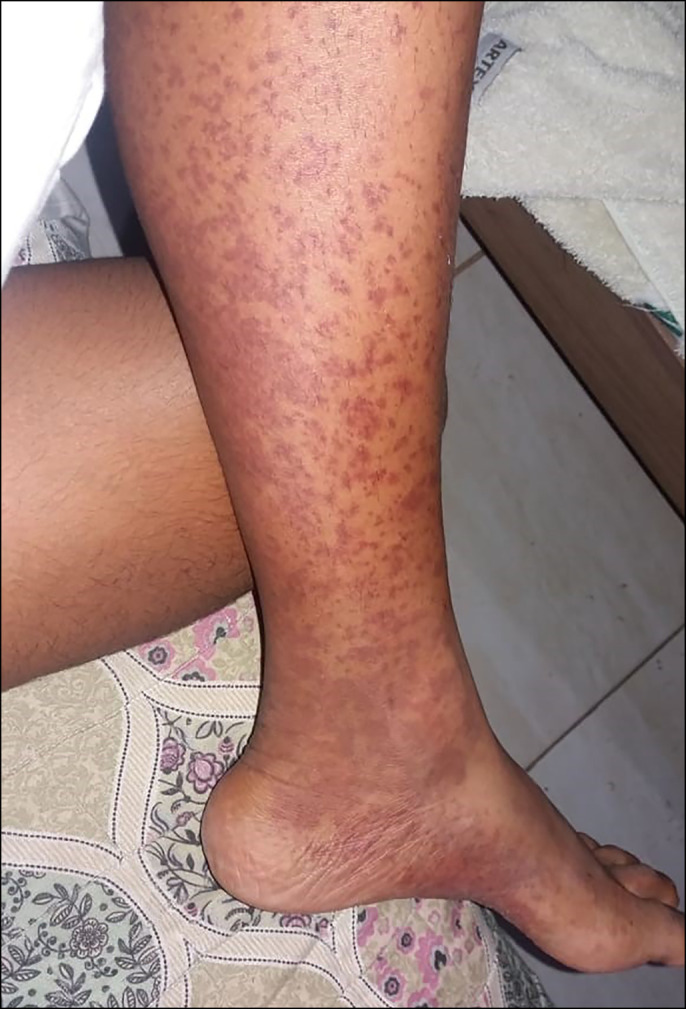
Purpuric lesions with areas of confluence in the lower extremities.

A 27-year-old woman was admitted to the hospital with a 6-month history of weakness, hyperchromic lesions in her legs, and arthralgia in her wrists and ankles. She had a rash and erythematous-violaceous purpura on the lower extremities on physical examination (Figure 1). The patient was previously healthy, with no personal or family history of autoimmune or liver diseases. She denied the use of alcohol or recent medications, transfusion of blood products, intravenous drug abuse, unprotected sex, tattoo, and previous infections.

On admission, blood count, kidney function, total bilirubin, alkaline phosphatase, albumin, and coagulation profile were normal. An increase in the erythrocyte sedimentation rate (ESR) of 56 mm/hr, in aspartate aminotransferase of 291 IU/L, and in alanine aminotransferase of 380 IU/L was found. She had no laboratory tests checked before hospital presentation. Because the patient had palpable purpura, arthralgia, and elevated ESR, the hypothesis of vasculitis was considered. The rheumatologic panel showed rheumatoid factor 201 IU/L (reference: <30 IU/L), ANA 1/160, normal C3, and a low C4 of 0.07 g/L (reference: 0.1–0.4 g/L). Anti-DNA, anti-SM, P-ANCA, and C-ANCA were negative, whereas the laboratory test result was positive for mixed cryoglobulins (IgM-IgG) in cryoprecipitate by immunoelectrophoresis. A clinical diagnosis of CV was made, and a skin biopsy was performed. A histopathological study showed leukocytoclastic cutaneous vasculitis, also consistent with CV diagnosis.

**Figure 2.**
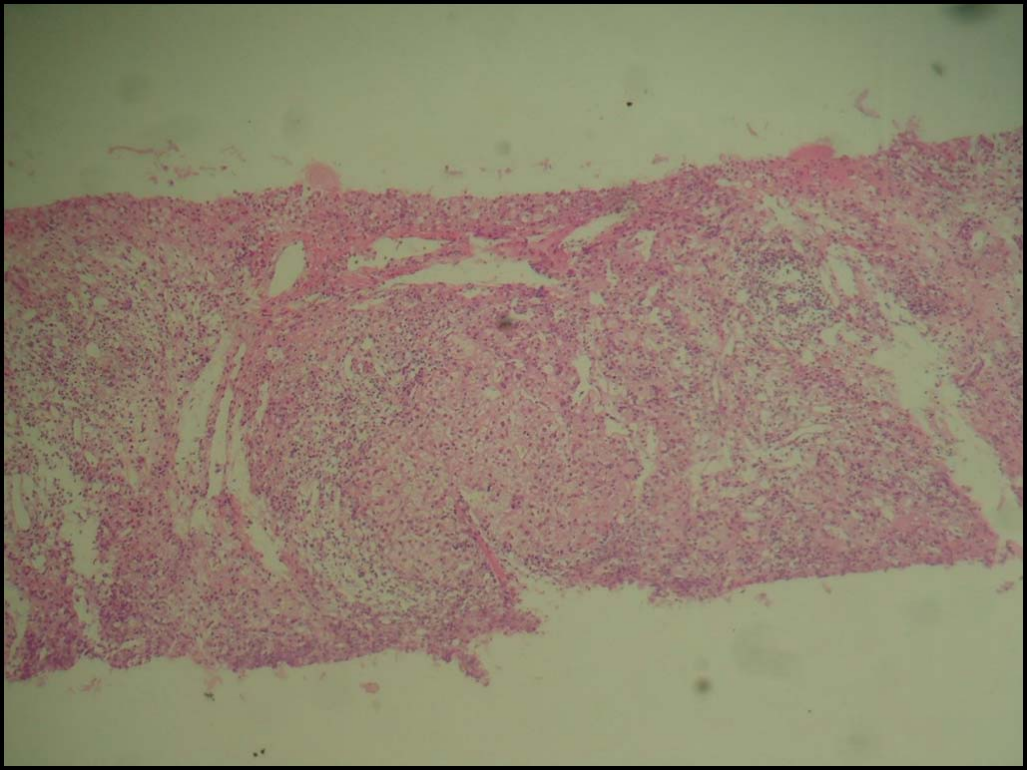
Histopathology of the liver with areas of parenchymal collapse and intense lobular lymphoplasmacytic inflammation (hematoxylin and eosin stain, ×20).

In view of the high levels of aminotransferases and the important association of HCV with cryoglobulinemia, an anti-HCV test resulted negative. Owing to the possibility of a false-negative anti-HCV, the HCV RNA was investigated, and it was negative in plasma and cryoprecipitate. Serologic testing was consistent with immunity because of hepatitis B vaccination (HBsAg negative, anti-HBs positive, and anti-HBc negative). Right upper quadrant ultrasound showed no abnormalities. The AIH diagnosis was considered because it was a young female patient with elevated aminotransferases, negative serology for hepatitis, no history of alcohol intake, and positive antinuclear antibody (ANA). Therefore, immunoglobulin levels were measured, and the IgG value was 39.3 g/L (7.0–16.0 g/L). Anti-LKM1 and anti-smooth muscle antibodies were not reactive. Liver biopsy showed lymphoplasmacytic infiltrate compatible with AIH (Figure 2).

Rituximab was not available at the hospital to initiate treatment for CV. A treatment regimen was initiated for both conditions with prednisone 1 mg/kg and azathioprine 3 mg/kg. The patient presented with regression of cutaneous vasculitis and decreased aminotransferases (aspartate aminotransferase 15 IU/L and alanine aminotransferase 13 IU/L) and ESR (32 mm in the first hour) within 30 days of therapy. Azathioprine was maintained, and a gradual corticosteroid reduction was performed. Prednisone 60 mg/d was given for 3 months, then reduced 10 mg per month to 30 mg/d, and then tapered off gradually. After 1 year, the patient no longer required the use of prednisone.

## DISCUSSION

The association of cryoglobulinemia with HCV is well documented. Such infection represents the etiology of this vasculitis in approximately 80% of the cases.^2^ HCV infection should always be investigated in patients with CV and elevated aminotransferases. The investigation should be conducted with anti-HCV antibodies and HCV RNA in serum and in cryoprecipitate because detection of HCV RNA in cryoprecipitate is the most sensitive method.^6^ In this case report, all tests for hepatitis C were negative, so it was a challenge to identify the etiology of liver involvement for this patient. The association of cryoglobulinemia with other causes of chronic liver disease, such as hepatitis B and alcohol, is uncommon.^7^ Trejo et al evaluated 443 patients with cryoglobulinemia and found 331 patients (75%) with infectious diseases, almost all of them with hepatitis C. The authors identified no patients with AIH.^8^

AIH is a disease more prevalent in young women that leads to chronic inflammatory liver damage.^9^ Extrahepatic autoimmune diseases are described in 20%–50% of patients with AIH, including autoimmune thyroiditis, celiac disease, inflammatory bowel disease, rheumatoid arthritis, and Sjogren syndrome.^10^ Among autoimmune skin diseases, the most frequent association is with alopecia, vitiligo, and psoriasis.^11^ A systematic review of skin manifestations associated with AIH performed in 2017 identified only 7 cases of cutaneous vasculitis, similar to the present case. However, none of the 7 cases was CV.^12^

Floreani et al conducted a study with 73 patients diagnosed with type I AIH and identified only 1 patient with cryoglobulinemia.^13^ However, there are no details on the clinical presentation of CV in this patient. We identified only 3 other cases of the association of CV with AIH. In one of them, a 73-year-old man had palpable purpura in the lower limbs and an increase in aminotransferases, very similar to this case reported.^14^ Biecker et al reported another case in which celiac disease, a recognized trigger for autoimmune disorders, was associated with AIH and CV.^15^ Evans et al also reported a case of AIH with cryoglobulinemia, where vasculitis led to renal involvement with glomerulonephritis and anuric acute kidney injury.^16^

We present a patient with CV with hepatic involvement not associated with HCV. Laboratory evaluation and liver biopsy were essential for the diagnosis of AIH. This report alerts physicians that despite a rare association, AIH should be remembered in patients with HCV-negative cryoglobulinemia.

## DISCLOSURES

Author contributions: PAdC Gouveia wrote and edited the article and is the article guarantor. MTSP Leal wrote the article. S. Rampche revised the article for intellectual content.

Financial disclosure: None to report.

Previous presentation: This case was previously presented at 15th Brazilian Congress of Internal Medicine; 2019.

Informed consent was obtained for this case report.
